# (*E*)-2-({2-[(*E*)-(Hy­droxy­imino)­meth­yl]phen­oxy}meth­yl)-3-*p*-tolyl­acrylonitrile

**DOI:** 10.1107/S160053681200270X

**Published:** 2012-02-04

**Authors:** G. Suresh, V. Sabari, J. Srinivasan, Bakthadoss Mannickam, S. Aravindhan

**Affiliations:** a Department of Physics, Presidency College, Chennai 600 005, India; b Department of Organic Chemistry, University of Madras, Chennai 600 025, India

## Abstract

In the title compound, C_18_H_16_N_2_O_2_, the hy­droxy­ethanimine group is essentially coplanar with the ring to which it is attached (C—C—N—O torsion angle = −176.9°). Mol­ecules are linked into cyclic centrosymmetric *R*_2_^2^(6) dimers *via* O—H⋯N hydrogen bonds.

## Related literature

For the structures of other acrylate derivatives, see: Zhang *et al.* (2009 ▶); Wang *et al.* (2011 ▶); SakthiMurugesan *et al.* (2011 ▶); Govindan *et al.* (2011 ▶). For the use of oxime ligands in coordination chemistry, see: Chaudhuri (2003 ▶). For the biological activity of caffeic acids, see: Hwang *et al.* (2001 ▶); Altug *et al.* (2008 ▶); Ates *et al.* (2006 ▶); Atik *et al.* (2006 ▶); Padinchare *et al.* (2001 ▶).
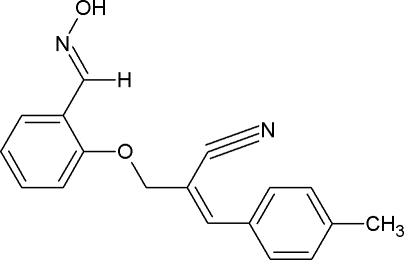


## Experimental

### 

#### Crystal data

C_18_H_16_N_2_O_2_
*M* *_r_* = 292.33
Triclinic, 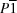

*a* = 8.4851 (2) Å
*b* = 9.3900 (3) Å
*c* = 10.0779 (3) Å
α = 100.208 (2)°
β = 90.725 (1)°
γ = 105.206 (1)°
*V* = 761.10 (4) Å^3^
*Z* = 2
Mo *K*α radiation
μ = 0.08 mm^−1^
*T* = 293 K
0.2 × 0.2 × 0.2 mm

#### Data collection

Oxford Diffraction Xcalibur-S diffractometer
Absorption correction: multi-scan (*CrysAlis PRO*; Oxford Diffraction, 2009 ▶) *T*_min_ = 0.980, *T*_max_ = 0.990
18160 measured reflections
4229 independent reflections
3031 reflections with *I* > 2σ(*I*)
*R*_int_ = 0.022

#### Refinement

*R*[*F* ^2^ > 2σ(*F* ^2^)] = 0.047
*wR*(*F* ^2^) = 0.151
*S* = 1.03
4229 reflections
201 parameters
H-atom parameters constrained
Δρ_max_ = 0.24 e Å^−3^
Δρ_min_ = −0.18 e Å^−3^

### 

Data collection: *CrysAlis PRO* (Oxford Diffraction, 2009 ▶); cell refinement: *CrysAlis PRO*; data reduction: *CrysAlis PRO*; program(s) used to solve structure: *SHELXS97* (Sheldrick, 2008 ▶); program(s) used to refine structure: *SHELXL97* (Sheldrick, 2008 ▶); molecular graphics: *ORTEP-3 for Windows* (Farrugia, 1997 ▶); software used to prepare material for publication: *PLATON* (Spek, 2009 ▶).

## Supplementary Material

Crystal structure: contains datablock(s) I, global. DOI: 10.1107/S160053681200270X/bt5756sup1.cif

Structure factors: contains datablock(s) I. DOI: 10.1107/S160053681200270X/bt5756Isup2.hkl

Supplementary material file. DOI: 10.1107/S160053681200270X/bt5756Isup3.cml

Additional supplementary materials:  crystallographic information; 3D view; checkCIF report

## Figures and Tables

**Table 1:** Hydrogen-bond geometry (Å, °)

*D*—H⋯*A*	*D*—H	H⋯*A*	*D*⋯*A*	*D*—H⋯*A*
O1—H1*A*⋯N1^i^	0.82	2.10	2.795 (2)	143

Symmetry code: (i) 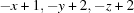.

**Fig. 1.**
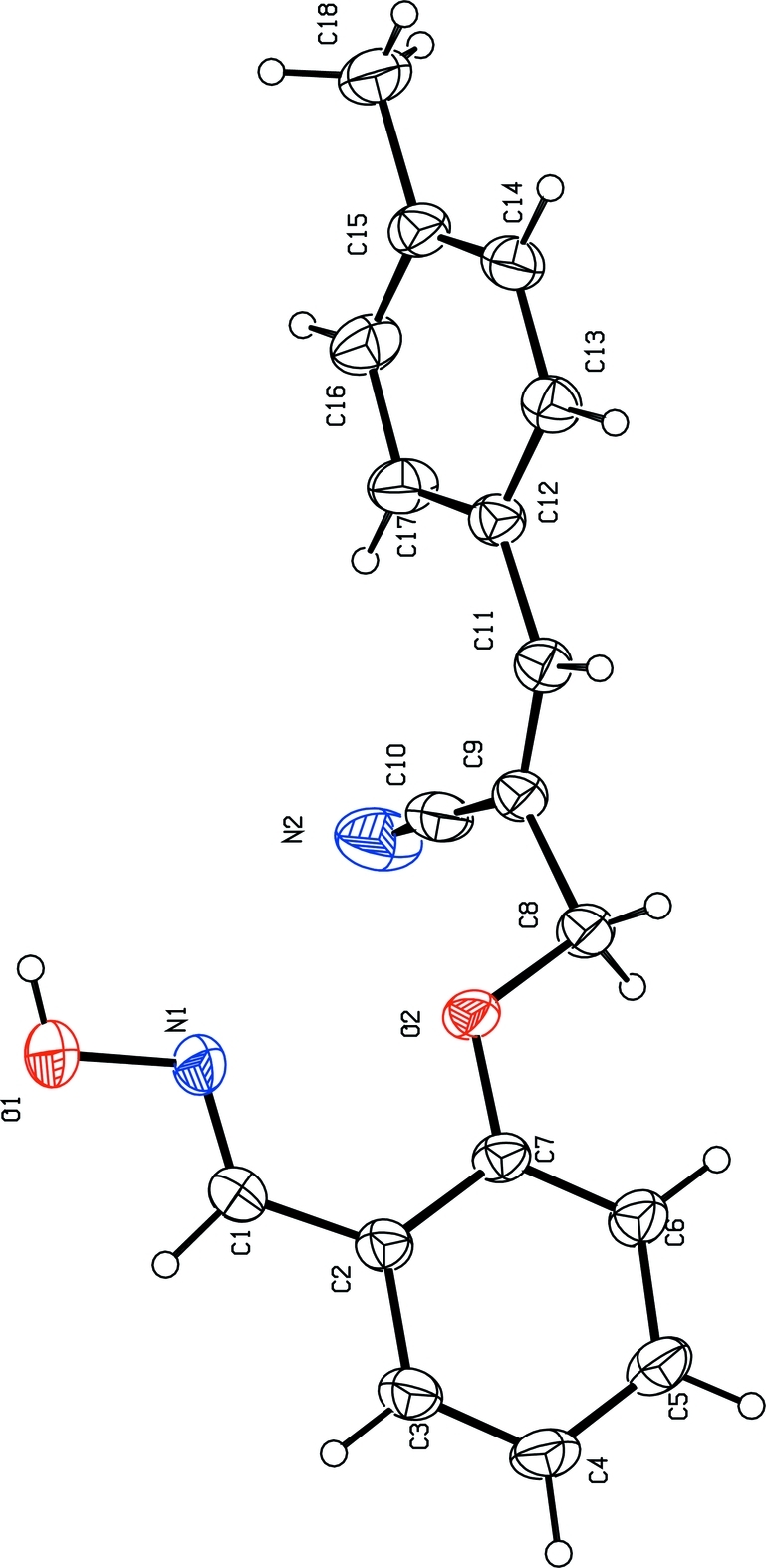
The molecular structure of the title compound, showing 30% probability displacement ellipsoids for non-H atoms.

**Fig. 2.**
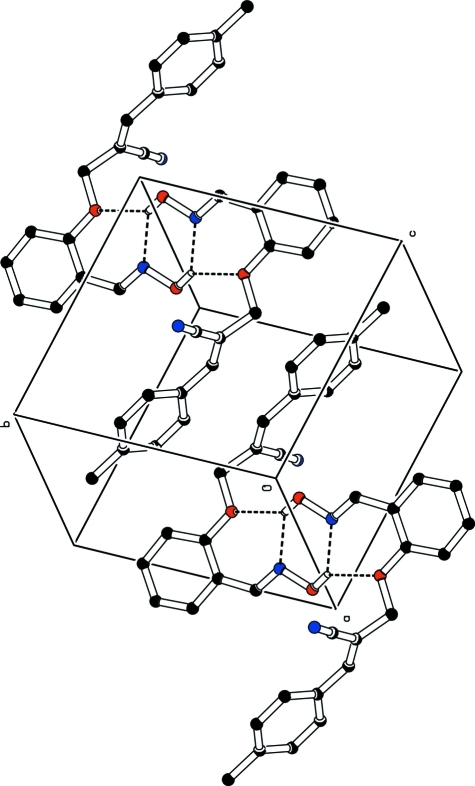
A view of the crystal packing. H atoms not involved in hydrogen bonding (dashed lines) have been omitted for clarity.

## supplementary crystallographic information

### Comment

Recently, 2-cyanoacrylates have been extensively used as agrochemicals because of their unique mechanism of action and good environmental profiles (Zhang *et al.*, 2009). Oximes are a classical type of chelating ligands which are widely used in coordination and analytical chemistry (Chaudhuri, 2003).Some naturally occurring caffeic acids and their esters attract much attention in biology and medicine (Hwang *et al.*, 2001; Altug *et al.*, 2008).These compounds show antiviral, antibacterial, vasoactive, antiatherogenic, antiproliferative, antioxidant and antiinflammatory properties (Atik *et al.*,2006; Padinchare *et al.*, 2001; Ates *et al.*, 2006). Against this background,and in order to obtain detailed information on molecular conformations in the solid state, an X-ray study of the title compound was carried out and the results are presented here. X-Ray analysis confirms the molecular structure and atom connectivity as illustrated in Fig. 1. The oxime group having the C=N forming an E configuration.the hydroxy ethanimine group is essentially coplanar with the ring to which it is attached.

The hydroxy ethanimine group in the molecules are linked into cyclic centrosymmetric dimers *via* O—H···N hydrogen bonds with the motif *R*_2_^2^(6) (Wang *et al.*, 2011; Govindan *et al.*, 2011; SakthiMurugesan *et al.*, 2011). The crystal packing is stabilized by intermolecular O—H···N hydrogen bonds (Fig. 2).

### Experimental

To a stirred solution of (*E*)-2-((2-formylphenoxy)methyl)-3-*p*-tolylacrylonitrile (4 mmol) in 10 ml of EtOH/H_2_O mixture (1:1) was added NH_2_OH.HCl (6 mmol) in the presence of 50% NaOH at room temperature. Then the reaction mixture was allowed to stir at room temperature for 1.5 h. After completion of the reaction, solvent was removed and the crude mass was diluted with water (15 ml) and extracted with ethyl acetate (3 τimes 15 ml). The combined organic layer was washed with brine (2 τimes 10 ml) and dried over anhydrous Na_2_SO_4_ and then evaporated under reduced pressure to obtain (*E*)-2-((2-((*E*)-(Hydroxyimino)methyl)phenoxy)methyl)-3-*p*-tolylacrylonitrile as a colourless solid.

### Refinement

H atoms were found in a difference map but treated as riding with O-H = 0.82Å, and C-H = 0.93-0.97Å. U(H) was set to 1.5 U_eq_(O, C_methyl_) or 1.2 U_eq_(C)

### Crystal data

**Table:**

C_18_H_16_N_2_O_2_	*Z* = 2
*M**_r_* = 292.33	*F*(000) = 308
Triclinic, *P*1	*D*_x_ = 1.276 Mg m^−^^3^
Hall symbol: -P 1	Mo *K*α radiation, λ = 0.71073 Å
*a* = 8.4851 (2) Å	Cell parameters from 8725 reflections
*b* = 9.3900 (3) Å	θ = 2.8–29.1°
*c* = 10.0779 (3) Å	µ = 0.08 mm^−^^1^
α = 100.208 (2)°	*T* = 293 K
β = 90.725 (1)°	Triclinic, colourless
γ = 105.206 (1)°	0.2 × 0.2 × 0.2 mm
*V* = 761.10 (4) Å^3^	

### Data collection

**Table:**

Oxford Diffraction Xcalibur-S diffractometer	4229 independent reflections
Radiation source: fine-focus sealed tube	3031 reflections with *I* > 2σ(*I*)
Graphite monochromator	*R*_int_ = 0.022
Detector resolution: 15.9948 pixels mm^-1^	θ_max_ = 29.6°, θ_min_ = 2.1°
ω scans	*h* = −11→11
Absorption correction: multi-scan (*CrysAlis PRO*; Oxford Diffraction, 2009)	*k* = −12→12
*T*_min_ = 0.980, *T*_max_ = 0.990	*l* = −13→13
18160 measured reflections	

### Refinement

**Table:**

Refinement on *F*^2^	Secondary atom site location: difference Fourier map
Least-squares matrix: full	Hydrogen site location: inferred from neighbouring sites
*R*[*F*^2^ > 2σ(*F*^2^)] = 0.047	H-atom parameters constrained
*wR*(*F*^2^) = 0.151	*w* = 1/[σ^2^(*F*_o_^2^) + (0.0732*P*)^2^ + 0.135*P*] where *P* = (*F*_o_^2^ + 2*F*_c_^2^)/3
*S* = 1.03	(Δ/σ)_max_ = 0.001
4229 reflections	Δρ_max_ = 0.24 e Å^−^^3^
201 parameters	Δρ_min_ = −0.18 e Å^−^^3^
0 restraints	Extinction correction: *SHELXL97* (Sheldrick, 2008)
Primary atom site location: structure-invariant direct methods	Extinction coefficient: 0.0173 (18)

### Special details

**Table:**

Geometry. All e.s.d.'s (except the e.s.d. in the dihedral angle between two l.s. planes) are estimated using the full covariance matrix. The cell e.s.d.'s are taken into account individually in the estimation of e.s.d.'s in distances, angles and torsion angles; correlations between e.s.d.'s in cell parameters are only used when they are defined by crystal symmetry. An approximate (isotropic) treatment of cell e.s.d.'s is used for estimating e.s.d.'s involving l.s. planes.
Refinement. Refinement of *F*^2^ against ALL reflections. The weighted *R*-factor *wR* and goodness of fit *S* are based on *F*^2^, conventional *R*-factors *R* are based on *F*, with *F* set to zero for negative *F*^2^. The threshold expression of *F*^2^ > σ(*F*^2^) is used only for calculating *R*-factors(gt) *etc*. and is not relevant to the choice of reflections for refinement. *R*-factors based on *F*^2^ are statistically about twice as large as those based on *F*, and *R*- factors based on ALL data will be even larger.

### Fractional atomic coordinates and isotropic or equivalent isotropic displacement parameters (Å^2^)

**Table:**

	*x*	*y*	*z*	*U*_iso_*/*U*_eq_	
C1	0.2633 (2)	0.81093 (18)	1.11597 (16)	0.0584 (4)	
H1	0.2024	0.8544	1.1791	0.070*	
C2	0.22978 (17)	0.64825 (16)	1.09291 (13)	0.0453 (3)	
C3	0.13864 (19)	0.57501 (19)	1.18740 (15)	0.0561 (4)	
H3	0.1003	0.6318	1.2585	0.067*	
C4	0.10362 (19)	0.42252 (19)	1.17926 (16)	0.0589 (4)	
H4	0.0403	0.3767	1.2425	0.071*	
C5	0.1631 (2)	0.33864 (18)	1.07685 (16)	0.0586 (4)	
H5	0.1429	0.2356	1.0721	0.070*	
C6	0.25320 (19)	0.40619 (16)	0.98021 (14)	0.0532 (3)	
H6	0.2938	0.3482	0.9115	0.064*	
C7	0.28343 (16)	0.55934 (15)	0.98496 (12)	0.0421 (3)	
C8	0.3950 (2)	0.53835 (16)	0.76860 (13)	0.0522 (3)	
H8A	0.3004	0.4535	0.7396	0.063*	
H8B	0.4874	0.5003	0.7866	0.063*	
C9	0.43216 (17)	0.63100 (15)	0.66042 (12)	0.0450 (3)	
C10	0.29368 (19)	0.66178 (18)	0.60167 (15)	0.0559 (4)	
C11	0.58348 (17)	0.67335 (15)	0.61876 (13)	0.0460 (3)	
H11	0.6609	0.6433	0.6650	0.055*	
C12	0.64990 (16)	0.75770 (15)	0.51448 (12)	0.0435 (3)	
C13	0.81374 (17)	0.77192 (17)	0.48925 (14)	0.0495 (3)	
H13	0.8746	0.7272	0.5380	0.059*	
C14	0.88750 (18)	0.85082 (17)	0.39364 (15)	0.0549 (4)	
H14	0.9974	0.8589	0.3795	0.066*	
C15	0.80160 (19)	0.91823 (16)	0.31820 (14)	0.0536 (4)	
C16	0.6389 (2)	0.9040 (2)	0.34270 (17)	0.0634 (4)	
H16	0.5787	0.9487	0.2933	0.076*	
C17	0.56333 (19)	0.8258 (2)	0.43805 (17)	0.0599 (4)	
H17	0.4535	0.8182	0.4517	0.072*	
C18	0.8817 (3)	1.0064 (2)	0.21478 (18)	0.0740 (5)	
H18A	0.8424	0.9511	0.1258	0.111*	
H18B	0.9982	1.0232	0.2247	0.111*	
H18C	0.8558	1.1013	0.2278	0.111*	
N1	0.36637 (16)	0.89828 (13)	1.05886 (12)	0.0535 (3)	
N2	0.1784 (2)	0.6810 (2)	0.55663 (18)	0.0851 (5)	
O1	0.37363 (18)	1.04895 (13)	1.10956 (14)	0.0795 (4)	
H1A	0.4451	1.1035	1.0733	0.119*	
O2	0.36281 (13)	0.63175 (10)	0.88863 (9)	0.0495 (3)	

### Atomic displacement parameters (Å^2^)

**Table:**

	*U*^11^	*U*^22^	*U*^33^	*U*^12^	*U*^13^	*U*^23^
C1	0.0724 (10)	0.0543 (9)	0.0521 (8)	0.0226 (7)	0.0238 (7)	0.0100 (7)
C2	0.0489 (7)	0.0495 (7)	0.0402 (6)	0.0151 (6)	0.0072 (5)	0.0123 (5)
C3	0.0607 (8)	0.0672 (10)	0.0479 (7)	0.0241 (7)	0.0193 (6)	0.0195 (7)
C4	0.0598 (9)	0.0687 (10)	0.0552 (8)	0.0151 (7)	0.0152 (7)	0.0322 (8)
C5	0.0720 (10)	0.0498 (8)	0.0534 (8)	0.0072 (7)	0.0059 (7)	0.0218 (7)
C6	0.0708 (9)	0.0444 (8)	0.0419 (7)	0.0102 (7)	0.0085 (6)	0.0091 (6)
C7	0.0475 (6)	0.0442 (7)	0.0330 (6)	0.0075 (5)	0.0036 (5)	0.0102 (5)
C8	0.0743 (9)	0.0423 (7)	0.0355 (6)	0.0098 (6)	0.0139 (6)	0.0035 (5)
C9	0.0583 (8)	0.0414 (7)	0.0322 (6)	0.0099 (6)	0.0098 (5)	0.0037 (5)
C10	0.0535 (8)	0.0653 (10)	0.0487 (8)	0.0125 (7)	0.0183 (6)	0.0147 (7)
C11	0.0536 (7)	0.0481 (7)	0.0361 (6)	0.0132 (6)	0.0034 (5)	0.0078 (5)
C12	0.0467 (7)	0.0444 (7)	0.0368 (6)	0.0085 (5)	0.0052 (5)	0.0059 (5)
C13	0.0479 (7)	0.0519 (8)	0.0467 (7)	0.0115 (6)	0.0030 (5)	0.0064 (6)
C14	0.0498 (7)	0.0533 (8)	0.0547 (8)	0.0060 (6)	0.0144 (6)	0.0031 (7)
C15	0.0655 (9)	0.0440 (7)	0.0430 (7)	0.0021 (6)	0.0115 (6)	0.0042 (6)
C16	0.0644 (9)	0.0693 (11)	0.0619 (9)	0.0150 (8)	0.0042 (7)	0.0307 (8)
C17	0.0475 (7)	0.0738 (11)	0.0657 (9)	0.0170 (7)	0.0114 (7)	0.0310 (8)
C18	0.0942 (13)	0.0603 (10)	0.0604 (10)	0.0033 (9)	0.0250 (9)	0.0171 (8)
N1	0.0721 (8)	0.0417 (6)	0.0470 (6)	0.0179 (6)	0.0113 (6)	0.0043 (5)
N2	0.0583 (9)	0.1209 (15)	0.0875 (11)	0.0292 (9)	0.0200 (8)	0.0404 (11)
O1	0.1108 (11)	0.0418 (6)	0.0846 (9)	0.0226 (6)	0.0388 (8)	0.0032 (6)
O2	0.0706 (6)	0.0383 (5)	0.0342 (4)	0.0058 (4)	0.0158 (4)	0.0051 (4)

### Geometric parameters (Å, º)

**Table:**

C1—N1	1.2541 (19)	C10—N2	1.143 (2)
C1—C2	1.454 (2)	C11—C12	1.4578 (18)
C1—H1	0.9300	C11—H11	0.9300
C2—C3	1.3963 (19)	C12—C13	1.3923 (19)
C2—C7	1.4063 (17)	C12—C17	1.396 (2)
C3—C4	1.371 (2)	C13—C14	1.376 (2)
C3—H3	0.9300	C13—H13	0.9300
C4—C5	1.369 (2)	C14—C15	1.382 (2)
C4—H4	0.9300	C14—H14	0.9300
C5—C6	1.385 (2)	C15—C16	1.382 (2)
C5—H5	0.9300	C15—C18	1.504 (2)
C6—C7	1.3850 (19)	C16—C17	1.376 (2)
C6—H6	0.9300	C16—H16	0.9300
C7—O2	1.3670 (15)	C17—H17	0.9300
C8—O2	1.4371 (15)	C18—H18A	0.9600
C8—C9	1.4985 (19)	C18—H18B	0.9600
C8—H8A	0.9700	C18—H18C	0.9600
C8—H8B	0.9700	N1—O1	1.4013 (15)
C9—C11	1.3371 (19)	O1—H1A	0.8200
C9—C10	1.427 (2)		
			
N1—C1—C2	126.24 (13)	C9—C11—C12	132.12 (13)
N1—C1—H1	116.9	C9—C11—H11	113.9
C2—C1—H1	116.9	C12—C11—H11	113.9
C3—C2—C7	117.57 (13)	C13—C12—C17	117.22 (13)
C3—C2—C1	116.91 (12)	C13—C12—C11	117.23 (12)
C7—C2—C1	125.51 (12)	C17—C12—C11	125.54 (12)
C4—C3—C2	122.44 (14)	C14—C13—C12	121.36 (13)
C4—C3—H3	118.8	C14—C13—H13	119.3
C2—C3—H3	118.8	C12—C13—H13	119.3
C5—C4—C3	119.14 (13)	C13—C14—C15	121.31 (13)
C5—C4—H4	120.4	C13—C14—H14	119.3
C3—C4—H4	120.4	C15—C14—H14	119.3
C4—C5—C6	120.48 (14)	C16—C15—C14	117.50 (14)
C4—C5—H5	119.8	C16—C15—C18	120.80 (16)
C6—C5—H5	119.8	C14—C15—C18	121.69 (15)
C7—C6—C5	120.58 (13)	C17—C16—C15	121.93 (15)
C7—C6—H6	119.7	C17—C16—H16	119.0
C5—C6—H6	119.7	C15—C16—H16	119.0
O2—C7—C6	123.63 (12)	C16—C17—C12	120.67 (14)
O2—C7—C2	116.70 (11)	C16—C17—H17	119.7
C6—C7—C2	119.66 (12)	C12—C17—H17	119.7
O2—C8—C9	108.34 (11)	C15—C18—H18A	109.5
O2—C8—H8A	110.0	C15—C18—H18B	109.5
C9—C8—H8A	110.0	H18A—C18—H18B	109.5
O2—C8—H8B	110.0	C15—C18—H18C	109.5
C9—C8—H8B	110.0	H18A—C18—H18C	109.5
H8A—C8—H8B	108.4	H18B—C18—H18C	109.5
C11—C9—C10	123.51 (12)	C1—N1—O1	111.61 (12)
C11—C9—C8	121.38 (13)	N1—O1—H1A	109.5
C10—C9—C8	115.02 (12)	C7—O2—C8	116.51 (10)
N2—C10—C9	176.93 (17)		
			
N1—C1—C2—C3	165.75 (16)	C8—C9—C11—C12	177.74 (13)
N1—C1—C2—C7	−13.2 (3)	C9—C11—C12—C13	−174.78 (14)
C7—C2—C3—C4	1.3 (2)	C9—C11—C12—C17	5.4 (2)
C1—C2—C3—C4	−177.81 (15)	C17—C12—C13—C14	0.5 (2)
C2—C3—C4—C5	1.6 (2)	C11—C12—C13—C14	−179.33 (12)
C3—C4—C5—C6	−2.0 (2)	C12—C13—C14—C15	−0.4 (2)
C4—C5—C6—C7	−0.5 (2)	C13—C14—C15—C16	0.3 (2)
C5—C6—C7—O2	−175.94 (13)	C13—C14—C15—C18	179.17 (14)
C5—C6—C7—C2	3.5 (2)	C14—C15—C16—C17	−0.2 (2)
C3—C2—C7—O2	175.69 (12)	C18—C15—C16—C17	−179.07 (16)
C1—C2—C7—O2	−5.3 (2)	C15—C16—C17—C12	0.2 (3)
C3—C2—C7—C6	−3.8 (2)	C13—C12—C17—C16	−0.4 (2)
C1—C2—C7—C6	175.23 (14)	C11—C12—C17—C16	179.43 (15)
O2—C8—C9—C11	108.86 (14)	C2—C1—N1—O1	−176.87 (15)
O2—C8—C9—C10	−74.60 (16)	C6—C7—O2—C8	8.7 (2)
C11—C9—C10—N2	156 (3)	C2—C7—O2—C8	−170.73 (12)
C8—C9—C10—N2	−21 (3)	C9—C8—O2—C7	162.41 (12)
C10—C9—C11—C12	1.5 (2)		

### Hydrogen-bond geometry (Å, º)

**Table:**

*D*—H···*A*	*D*—H	H···*A*	*D*···*A*	*D*—H···*A*
O1—H1*A*···N1^i^	0.82	2.10	2.795 (2)	143

Symmetry code: (i) −*x*+1, −*y*+2, −*z*+2.
